# Strengthening the connection between clinical research and clinical practice of cognitive rehabilitation

**DOI:** 10.3389/fresc.2023.1084071

**Published:** 2023-07-05

**Authors:** Nancy Chiaravalloti, Aubree Alexander

**Affiliations:** ^1^ Center for Neuropsychology and Neuroscience Research, Kessler Foundation, East Hanover, NJ, United States; ^2^ Department of Physical Medicine and Rehabilitation, Rutgers–New Jersey Medical School, Newark, NJ, United States

**Keywords:** cognitive rehabiltation, translation, evidence based research, cognition, implementation science

The translation of evidence based practice to clinical care in rehabilitation settings has been limited (1, 2). It can be argued that the research laboratory and the clinical care environment are dichotomous settings, substantially impeding the translation of research evidence to clinical care and the operationalization of aspects of the clinical environment to fit the demands of an empirical research study. While the research lab is characterized by high levels of control and structure, the clinical care environment is often fluid and unpredictable. Patient-centered care requires clinical flexibility and responsiveness to individual circumstances, especially in the context of cognitive rehabilitation. For example, when delivering cognitive rehabilitation, planned treatment interventions may need quick and creative alterations to address unexpected circumstances and/or fluctuations in fatigue, mood, behavior, or cognition that present over the course of a treatment session. In addition, clinical cognitive rehabilitation treatment plans are typically developed based on data obtained through formal evaluation, such as a neuropsychological assessment. Such treatment plans are individualized based on specific cognitive profiles and patterns of strengths and weaknesses. Treatment materials and activities are typically customized to suit individualized treatment needs. Furthermore, contextual and person-centered goals often form the backdrop for therapeutic interventions and clinicians consider varying individual factors, characteristics, and preferences to develop relevant, functional, and motivating plans of care (3, 4). Essential to this process is flexibility to address individual factors and treatment priorities. Therefore, cognitive rehabilitation interventions are most beneficial when they contain inherent flexibility and are easily modified to meet a range of clinical needs. However, flexible interventions are difficult to evaluate empirically within controlled research studies and it is thus challenging to build an evidence base for cognitive rehabilitation interventions with the flexibility necessary for clinical application.

Despite such challenges, the effective implementation of evidence based cognitive rehabilitation treatments is essential and the process of clinical implementation for cognitive rehabilitation has been specifically investigated (5–7). In an effort to examine the process of implementing cognitive rehabilitation intervention for dementia, one study allowed for flexible application of elements of the intervention depending upon individual needs, preferences, and contexts (5, 6). Documenting the process of intervention delivery allowed for greater understanding of real-world implementation and the ways in which therapists drew upon their professional experience and training to determine needed adaptations (5). Another study, involving implementation of a cognitive intervention for dementia in a community setting, provided pre-implementation training to maximize translation to the “real-world environment” (7). Prior to implementation of the intervention, providers were trained in how to adapt the demands of the cognitive tasks to align with an individual's cognitive skills and were provided with knowledge and skills training related to the cognitive intervention and cognitive demands of activities. During the course of the intervention, conferences were held between researchers and providers to discuss implementation and methods of adjusting activities to address different cognitive levels (7). While these studies document the need for flexibility in clinical applications for dementia specifically, such flexibility and adaptation are necessary across populations. These studies also indicate that the ability to work with the patient “off-manual” is essential for any cognitive rehabilitation program. Pre-implementation activities shown to be essential to the application of the cognitive rehabilitation protocol to the clinical environment included additional training of clinicians in protocol adaptation and pre-implementation conferences between clinicians and researchers. Furthermore, continued communication between clinicians and researchers throughout the implementation process was integral to administering flexible interventions.

In contrast to such pre-determined adaptations, it is more likely that modifications to evidence-based interventions happen spontaneously during an intervention session and are based on clinical judgment and skill, as well as patient needs and preferences. Knowledge obtained through clinical experience brings confidence and comfort in practice (8), which likely contributes to increased comfort with adapting intervention protocols. Less experienced clinicians may thus need increased training to effectively deliver flexible care (5). As noted, opportunities for training and consultation related to potential task modifications *both before and during* the implementation of the intervention are integral to delivering flexible interventions in practice. Additional modifications that may occur in clinical practice include applying an intervention to a clinical population that was not included in the empirical research or to an individual who may not have met inclusion criteria in the research studies associated with the intervention (9). For example, in many research studies individuals with multiple diagnoses are excluded from participation, yet in clinical practice multiple diagnoses often need to be addressed and managed to deliver care. Furthermore, modifications may also be needed to address organizational or institutional demands such as adjusting session duration and/or number of sessions, or modifying materials used in the intervention depending on resources available in clinical settings. Formally tracking and considering such modifications is an important step to quantifying and understanding their effects on outcomes (For more details see 10–12).

Given that cognitive rehabilitation is often delivered by professionals from varied clinical backgrounds, modifications may vary by discipline, and such differences will provide valuable information to researchers. In addition, researchers must be mindful of the need for multidisciplinary access to evidence-based cognitive rehabilitation interventions, as well as interdisciplinary education/training and collaboration (13–16). In such cases common language elements and shared terminology, as well as concrete examples of discipline specific language are crucial to enhance communication (15). A theoretical framework such as the *Rehabilitation Treatment Specification System* allows for a common language to be used among multi-disciplinary clinicians, interdisciplinary treatment teams, patients/families, researchers, and clinical training programs (17–20). Such a system possesses the potential to inform treatment decisions based on 3 specific characteristics: (1) measurable treatment targets, (2) treatment ingredients (therapist activities to address the targets), and (3) mechanisms of action (how the ingredients change the target); and, can be applied across disciplines, treatment modalities, and treatment settings (18, 20). In the future more wide-spread knowledge and application of this system has the potential to vastly improve the adoption of evidence-based techniques into the clinical environment.

Multidisciplinary training of clinicians in evidence-based cognitive rehabilitation interventions is necessary to ensure broad based knowledge of the constructs addressed by the treatment protocol as well as the appropriate application of the treatment to various clinical populations and practice domains. A strong history of collaboration within multidisciplinary teams is indeed a great strength of the field of clinical rehabilitation and such interprofessional collaboration and communication can contribute to improved knowledge translation and implementation of cognitive rehabilitation interventions (9, 15). Multidisciplinary teams and interprofessional collaborations have in fact contributed to the development of clinical practice guidelines and standards in the area of cognitive rehabilitation (15, 21–23). However, increased feedback from front-line clinical staff regarding the use of research-based interventions in practice will allow for “two-way” communication to enhance evidence-based practice. Furthermore, real-time communication between researchers and clinicians will help to increase the understanding of the needs of clinical environment and the identification of solutions to contribute to implementation.

Given the critical nature of such communication, methods of communication must be critically examined for utility. Evidence based research and clinical practice guidelines are commonly disseminated through journals, websites, print and electronic distribution, computerized decision-support, and audit (24). Creative communication tools that can be used to enhance knowledge dissemination include illustrations, infographics, podcasts, blogs, social media, briefing papers, or board games (25). However, many of these methods may be considered forms of “one way” communication and several questions remain regarding the uptake of this material within clinical practice such as how well the guidelines are actually used in the clinical context, as well as whether clinical professionals are being reached effectively using these tools.

Rehabilitation professionals reportedly rely on a variety of sources to inform clinical practice, including the empirical literature (14, 26), textbooks (26), clinical practice guidelines within the population being treated as well as in similar populations (14, 26), internal departmental protocols (26), continuing education (14), consultation with colleagues (14), clinical experience (14) and professional association websites (26). Clinicians may also need to rely on expert opinion to inform practice in areas of emerging research where robust evidence is not yet established (14), or they may need to rely on their own knowledge gained and developed through clinical experience (8). Given that cognitive rehabilitation is delivered by professionals from multiple clinical disciplines, it may be necessary for clinicians to access literature from professional publications and resources that are not specifically linked to their area of clinical background. As researchers disseminate information related to evidence-based care it is important to evaluate the scope and reach of the publication and determine other effective means of knowledge translation. Given significant time limitations in clinical care settings, time to stay current on the existing literature and to attend continuing education seminars is often not protected or available.

The challenges inherent in translating clinical research to clinical care have been widely recognized through several recent developments. The field of *Implementation science* has substantially grown in recent years. This literature identifies multiple strategies to increase implementation of healthcare research into clinical practice including assessments of the feasibility of interventions and process-based analyses, as well as the engagement of stakeholders (9, 22, 23). Models and strategies of implementation science are vast and include attention to characteristics of the intervention, including considerations of how the intervention can be adapted, qualities of the organization in which the intervention is to be implemented, qualities of those who will use the intervention (including clinicians and consumers), methods of collaboration between researchers and stakeholders, meaningful engagement of stakeholders in all aspects of the research process, methods of implementation, characteristics and needs of local communities, as well as knowledge translation, administrative issues and funding sources (For more details see 24, 27–33).

Moving to the future, experts have identified several factors to be addressed in an effort to increase the implementation of evidence-based research in cognitive rehabilitation. A recent narrative review of clinical practice guidelines in moderate to severe traumatic brain injury suggests a need for increased inclusion of functional outcome measures in rehabilitation research and increased inclusion of patients and stakeholders in outcome selection (34). In this way, researchers can incorporate the opinions of patients and stakeholders in identifying the treatment targets that are important to them. In addition, results of an international survey of allied health professionals who deliver cognitive rehabilitation services indicated a need for increased clinician involvement in the development of clinical practice guidelines, as well as increased audit and qualitative exploration of clinical practice (26). That is, effective implementation of evidence-based practice to improve patient outcomes and quality of care requires increased engagement of treatment providers, consumers, healthcare administrators, and health care organizations (35). Such engagement will provide useful information to researchers about available resources and potential limitations of the clinician environment. As noted, researchers must also be mindful of clinicians' needs. That is, clinicians may need more information about responders vs. non-responders, optimal treatment dose, and cost and value of evidence-based practice in order to apply research-based evidence in practice (35). Indeed, the integration of treatment providers, consumers, healthcare administrators, and health care organizations as suggested would serve to increase the researchers' knowledge of clinician needs enabling future research to be designed to address such needs. Finally, publications of research results must include the relevant details in intervention descriptions to allow replication and clinical implementation. Currently such descriptions are variable and often inadequate (36). In the presence of word count limitations in research publications, web-based communications of such details are essential to promote clinical implementation.

Potential barriers on the part of clinicians to implementing evidence-based practice also need to be addressed. Such barriers include lack of time, limited staffing resources, clinical demands, lack of authority to change practices, and resistance to change (14, 37). There are several potential solutions that can be considered with different solutions being more viable at different institutions depending on the organizational structure, organization resources and existing staff training. Across organizational structures, however, it is essential that all organizations providing clinical services seek to provide protected time for necessary training and education of staff. Indeed, this is the only way staff can stay abreast of the developing literature and obtain the necessary training to implement the most recent treatment protocols. This protected time will aid in the ability of clinical staff to best serve their patients and maximize patient outcomes. While financial resources would be optimal to enable travel to educational activities, such travel is no longer absolutely essential. Education can now be provided through distance learning, zoom calls, and webinars. Additionally, researchers often seek to share their knowledge; inviting researchers to provide education to clinicians, whether *via* distance learning or in person is always encouraged. Finally, from the research side, the implementation of knowledge translation activities into the research process is essential to maximize translation of research into clinical settings. This includes dissemination through sources known to be accessed by clinicians as noted above—empirical literature, continuing education, and professional association websites. Some conferences are more clinically focused and such conferences should be sought out by clinical researchers to promote knowledge translation and collaboration across institutional and geographic boundaries. Clinical forums and online established methods of clinical training exist on which clinicians rely for new information. Researchers must not only agree to present when invited for such activities, but actively seek them out to provide education regarding their research program and findings. Active communication is key to effective translation and that communication is the responsibility of both parties—the clinician and the researcher.

While increased clinician/researcher communication can be a focus of implementation it is sometimes more appropriate for implementation to occur at an organizational wide level. For example, one knowledge translation initiative, targeted specifically at the inter-professional application of the Cognitive Orientation to daily Occupational Performance (CO-OP) approach in inpatient stroke rehabilitation, was conducted at five rehabilitation hospitals in Canada (38). In this case, knowledge translation consisted of a 2-day workshop, 4 months of implementation support, a consolidation session, and infrastructure support. A sustainability plan was also implemented. Importantly, prior to the knowledge translation activities, there was no evidence of clinical implementation, despite several studies demonstrating its benefits, including randomized controlled trials in inpatient rehabilitation and sub-acute rehabilitation. Post-intervention, however, evidence of utilization was present on 20% of reviewed medical charts. This demonstrates that implementation of evidence based treatment does not occur automatically, but rather reflects an active process requiring investment from program developers, clinicians, and administrators, and supported by the organizational structure (35).

It is likely that increased integration of implementation science and knowledge translation paradigms in the area of cognitive rehabilitation will allow for increased communication and collaboration between cognitive rehabilitation researchers and practitioners and will contribute to increased development of flexible evidence-based protocols that can flow from the research lab to the more fluid demands of the treatment environment. Such flexibility will allow for guided modifications to evidence-based cognitive rehabilitation treatment protocols and data driven application of cognitive rehabilitation which aligns with clinical recommendations. Formal tracking and documentation of needed and implemented modifications, as well as improved dialogue between researchers and clinicians are necessities. Such dialogue will encourage a common language through which to communicate about cognitive rehabilitation concepts (15) and to develop increasingly flexible interventions to address individual needs and cognitive presentations.
